# Energy-safety trade-offs differ between winter-adapted species of prey to drive distinct activity patterns

**DOI:** 10.1007/s00442-025-05797-y

**Published:** 2025-09-22

**Authors:** Shotaro Shiratsuru, William H. Karasov, Jonathan N. Pauli

**Affiliations:** Department of Forest and Wildlife Ecology, University of Wisconsin, Madison, WI USA

**Keywords:** Energetics, Predation risk, Predator–prey, *Lepus americanus*, *Erethizon dorsatum*

## Abstract

Energy acquisition and risk avoidance can drive prey activity, but mechanistic understanding of the process through which prey traits mediate this energy-safety trade-off is lacking. We analyzed daily activity in winter of two winter-adapted prey that differ in antipredator strategies and physiological constraints on activity: snowshoe hares (*Lepus americanus*) and North American porcupines (*Erethizon dorsatum*). First, we constructed an energetics-based model to predict daily activity time for each species based on temperature and corresponding mass-dependent thermoregulatory costs. We then examined what factors drove the deviations of observed activity from the model prediction based on energy balance, considering individual variation. Neither hares nor porcupines achieved energy balance when temperatures fell below the thermoneutral zone. Activity deviations of hares from energy balance were explained by environmental factors associated with risk and energetics, with nominal individual variation being exhibited. In contrast, activity deviations of porcupines were independent of the environmental factors, and porcupines exhibited pronounced individual variation in activity. Our findings highlight that antipredator strategies and physiological constraints on activity can mediate the behavioral energy-safety trade-off of prey. Notably, morphological protection and energy reserves facilitate behavioral flexibility by relaxing the energy-safety trade-off, whereas dependence on behavioral predator avoidance limits the behavioral flexibility of prey.

**Supplementary Information:**

The online version contains supplementary material available at 10.1007/s00442-025-05797-y.

## Introduction

Animals acquire energy from their environments for maintenance, growth, and reproduction (Yodzis and Innes 1992). Activity, which requires energy expenditure, is mandatory for energy acquisition. Animal activity is also an important driver of species interactions (Werner 1992), and thus, a mechanistic understanding of activity can provide insights into how environmental conditions are translated into energetic status of animals (Humphries and McCann 2014), species interactions, and community dynamics (Werner 1992). Energy maximization can be considered a primary driver of animal activity (Schoener 1971), but other fitness-enhancing behaviors (e.g., mating) affect behavioral decisions of animals whether to be active or inactive (Studd et al. 2020). Moreover, activity levels tend to be associated with heightened predation risk (Houston and McNamara 2014). Predator avoidance generally reduces animal activity as prey attempt to conceal and hide from predators (Lima and Dill 1990). This interplay between activity and energy-gaining behaviors with inactivity to avoid predators has been conceptualized as the food-safety trade-off (McNamara and Houston 1987). Previous studies have examined how environmental conditions that affect the energetics of prey (e.g., temperature; de Barros et al. 2010 or resource availability; Matassa et al. 2016) and predation risk interactively drive prey activity. However, it is poorly understood how physiological constraints on prey activity (e.g., possession/lack of energy reserve) mediate these behavioral decisions of prey. Furthermore, traits linked to antipredator strategies (e.g., morphological traits for physical protection) impact perceived risk and thereby shape prey activity (Wirsing et al. 2021) and resulting energy expenditure. These potential mechanisms by which prey traits affect their activity by mediating the energy-safety trade-off have not been explored in an inter-specific comparison using the common currency of energy.

Prey adopt diverse antipredator mechanisms; some species alter their behaviors such as habitat selection, vigilance (Creel 2018), or reduced activity (Lima and Dill 1990), while others rely on morphological defense, use of refuges, or crypsis (Werner and Peacor 2003; Mills et al. 2013). Prey species predominantly dependent on behavioral predator avoidance should prioritize risk avoidance over energy acquisition in activity (Smith et al. 2019). In contrast, prey species that have evolved morphological defenses (e.g., quills/spines or dermal plates; Caro 2005) should experience less predation risk while foraging (Wirsing et al. 2021), which can increase the importance of energy acquisition relative to risk avoidance. Indeed, previous studies found that morphologically defended prey are less likely to alter their behaviors under high predation risk (e.g., McLean and Godin 1989). In addition to species-specific differences, intra-specific individual variation in antipredator behaviors is common (Huntingford and Giles 1987; Mazza et al. 2019). The amount of individual variation in antipredator behaviors is mediated by behavioral patterns and life histories of the species, implying its important fitness and evolutionary consequences (Møller and Garamszegi 2012). However, how the primary antipredator strategies (e.g., behavioral vs morphological) of the species structure individual variation in antipredator behaviors, and more broadly, activity, remains unclear. Individual variation in activity associated with risk avoidance should be larger in prey dependent on non-behavioral antipredator strategies, given that relaxed perceived risk may enable flexible behavioral decisions (Wirsing et al. 2021).

Species traits linked to energetics are also expected to impact prey activity by mediating energy intake and expenditure during activity or energy requirement through foraging. Effects of ambient temperature on energy gain and efficiency are different between endotherms and ectotherms (Yodzis and Innes 1992; Humphries and Umbanhowar 2007), and morphological traits affect energetic costs of locomotion (Parker et al. 1984). In addition, species that accumulate circannual fat stores can minimize activity for extended durations (Coltrane et al. 2011); in contrast, species that do not rely on stored energy need to be more constantly active for foraging (Whittaker and Thomas 1983). The decision whether to be active or inactive under the energy-safety trade-off becomes pronounced in winter due to harsh environmental conditions. Low temperatures increase thermoregulatory costs (Irving et al. 1955), and snow increases locomotory costs (Parker et al. 1984). Resources are limited in winter especially for herbivorous prey (Coltrane and Barboza 2010). In addition to these energy deficits, prey are exposed to high predation pressure (Krebs et al. 2018), further tightening the behavioral energy-safety trade-off in winter (Oates et al. 2019). In addition, physiological constraints on activity can contribute to individual variations in prey activity. Prey lacking energy reserves should have less flexibility in making behavioral decisions whether to forage or not, likely leading to smaller individual variation in deviation of activity from maintaining energy balance. Therefore, monitoring activity patterns of different prey species along the environmental and risk gradients in winter will enable us to examine how species traits mediate the process in which maintenance of energy balance and risk avoidance drive prey activity.

Snowshoe hares (*Lepus americanus*) and North American porcupines (*Erethizon dorsatum*) are widely distributed winter-adapted herbivorous prey in northern North America and nocturnally active throughout the winter (Mabille et al. 2011; Studd et al. 2019). Both species feed on nutritionally poor resources during winter (Wolff 1978; Coltrane and Barboza 2010). It has been also reported that the energy-safety trade-off plays a role in structuring activity patterns of both hares (Hik 1995; Majchrzak et al. 2022) and porcupines (Sweitzer 1996; Pokallus and Pauli 2016) and their populations are likely limited by risk effects of predators (DeWitt et al. 2017; Krebs et al. 2018). Despite these functional similarities, however, they possess distinct strategies for predator avoidance. While hares primarily rely on behavioral alteration (Hik 1995; Majchrzak et al. 2022) and crypsis induced by seasonal coat color change (Mills et al. 2013) for predator avoidance, porcupines are morphologically defended by ~ 30,000 quills and rest in natural shelters (Roze 2009). This morphological protection makes porcupines inaccessible for many predators besides fishers (*Pekania pennanti*) (Pokallus and Pauli 2015), whereas hares are exposed to diverse predators (Krebs et al. 2018). As for winter energetics, porcupines partly rely on catabolism of fat reserves from preceding summer and fall and thus they only need to forage to obtain 75–90% of their daily maintenance energy requirement (Coltrane et al. 2011), whereas lack of fat reserves requires hares to forage more constantly (Whittaker and Thomas 1983). Overall, because of these trait dissimilarities, comparison of winter activity patterns between hares and porcupines will provide insights into understanding how species traits mediate the process in which the energy-safety trade-off structures prey activity.

To examine activity patterns of endothermic prey in winter, we analyzed daily activity data obtained from free-living hares and porcupines using tri-axial accelerometers. We hypothesized that maintenance of energy balance is the core driver of prey activity in winter and deviation of prey activity from maintaining energy balance is explained by predation risk. We further hypothesized that prey traits associated with antipredator strategies and physiological constraints on activity affect the balance of the behavioral energy-safety trade-off. To test our hypotheses, we used an energetics-based model to predict daily activity time of both species based on daily temperature and corresponding mass-dependent thermoregulatory costs, assuming that prey minimize daily activity time while still maintaining energy balance (Schoener 1971). We then examined what factors explained the deviations of observed activity time from model predictions, considering inter-individual variation. Specifically, we considered environmental factors such as wind speed, snow depth, and lunar luminosity associated with predation risk or/and energetics in the analysis of activity deviations. We predicted that deviations of activity time would be affected by these environmental factors in hares to a larger degree compared to porcupines, due to relaxed energy-safety trade-off for porcupines induced by morphological defense and energy reserve. We further predicted that this relaxed energy-safety trade-off would result in larger individual variations in activity patterns under the energy-safety trade-off in porcupines than in hares.

## Materials and methods

### Data collection

In winter (late December–mid-April) 2022 and 2023, we captured and monitored snowshoe hares in the Chequamegon National Forest Medford District (45.291° N, 90.517° W) and porcupines in the Sandhill Wildlife Area (44.307° N, 90.129° W) in Wisconsin, USA (Figure S1). These two sites are < 150 km apart and exhibit similar winter conditions and predator species composition. Hares are exposed to multiple predators including fisher, coyote (*Canis latrans*), bobcat (*Lynx rufus*), and red fox (*Vulpes vulpes*) (Litvaitis et al. 1984; Etcheverry et al. 2005), whereas fisher is the primary predator of porcupines (Pokallus and Pauli 2015).

We opportunistically live-trapped hares and porcupines using Tomahawk live-traps (Tomahawk Live Trap Co., Tomahawk, Wisconsin, USA), determining upon each capture sex, measuring body mass and marking individuals with ear-tags (National Band & Tag Co, Newport, Kentucky, USA) for hares and with PIT-tags (AVID Friendchip; AVID Identification Systems, Norco, CA) for porcupines. We deployed accelerometer collars (Axy-Trek Mini, Technosmart Europe Srl., Rome, Italy) with VHF transmitters (R1830, Advanced Telemetry Systems, Isanti, Minnesota, USA) on 29 hares (10 and 21 collar deployments in winter 2022 and 2023, respectively, with 2 hares monitored in both winters) and 21 porcupines (8 and 16 collar deployments in winter 2022 and 2023, respectively, with 3 porcupines monitored in both winters), to quantify their activity when released. Upon each collar deployment, we made sure that the total collar mass was < 5% of animal’s body mass. Accelerometers recorded body acceleration of animals along three axes (dorsoventral, anterior–posterior and lateral) at 1 Hz and 10 Hz frequency for hares and porcupines, respectively, with ± 8 g forces. Our methods were approved by the University of Wisconsin-Madison Institute for Animal Care and Use Committee (protocol A005849-R02).

We obtained meteorological data including wind speed, cloud coverage, and air temperature recorded as a part of the Automated Surface Observing Systems (ASOS) as well as daily snow depth from the weather station (~ 25–30 km) nearest to each study area. Detailed information on meteorological data collection and preparation for the subsequent analyses can be found in Methods S1.

### Behavioral classification

Using a hierarchal decision tree developed for hare accelerometer data, we classified the behavior of the hares into four categories based on summary statistics calculated over 4-s time window; resting, hopping, sprinting, and foraging (foraging defined as feeding and travel with a single hop; Studd et al. 2019). We considered hopping, sprinting, and foraging as an active state and resting as an inactive state for our analysis, and the decision tree we used has been validated for classifying an active state of hares with an accuracy of 97.0% (Studd et al. 2019).

For porcupines, we first recorded time-stamped behaviors of collared animals with a digital video camera either upon release or by locating them using VHF. We recorded a total of 15.32 h of video from the 21 collared porcupines, and porcupine behavior in the videos was classified into four categories: foraging (feeding with a small movement), climbing/descending (traveling on/in a tree), traveling on the ground, and resting. We then assigned observed behaviors to corresponding accelerometer data for subsequent behavioral classification. To classify accelerometer data from porcupines, we trained Random Forest algorithm using AcceleRater, an open-access web application (Resheff et al. 2014; see Methods S2 for details). Finally, using the trained Random Forest algorithm, we annotated the full porcupine accelerometer dataset (summarized in 10-s time windows) including data points without observed behavioral assignment. As with hares, we considered all the behavioral categories besides resting as an active state for our analysis.

### Energetics-based activity model

We constructed energetics-based models to predict how hares and porcupines adjust their daily activity time in response to variation in temperature while maintaining daily energy balance. Our models consider energy intake from foraging during the activity period, activity- and inactivity-related energy expenditure, and thermoregulatory costs (Humphries and Umbanhowar 2007; Studd et al. 2020). Based on previous findings, we assumed that hares adjust their daily activity time so that daily net energy gain (NEG) would be 0 (i.e., they achieve daily energy balance and do not store large amounts of energy in tissue or routinely catabolize tissue energy stores) (Whittaker and Thomas 1983; Ellsworth et al. 2016). In contrast, porcupines enter winter with fat energy stores and may forage to meet only 75–90% of their daily energy requirement as they can gain 10–25% of their daily maintenance energy requirement from fat catabolism (Coltrane and Barboza 2010; Coltrane et al. 2011). For both species, we assumed that animals minimize their activity time while still meeting these minimum daily energy requirements (Schoener 1971). We obtained or calculated all the parameter values used in the models (*italicized* in the equations below) for both species from previous literature, scaling energy intake and expenditure with body mass (Methods S3 and Table S2). These assumptions result in the general model structure as follows:

For hares, on a daily basis,

1 $${\text{NEG}}a{-}{\text{ EE}}i = \, 0{\text{ kJ}}$$

and for porcupines, on a daily basis,

2 $${\text{NEG}}a{-}{\text{ EE}}i = \, - {39}.{\text{8M}}^{{0.{75}}} {\text{kJ}}$$

where NEG*a* (in kJ) is metabolizable energy intake (*I*, in kJ/h active) minus energy expenditure, while in the active state (EE_*a*_, in kJ), EE*i* (in kJ) is the energy expenditure during inactivity and M is the body mass of the animal (kg). The right hand side of Eq. (2) assumes the porcupine’s foraging does not meet its daily requirement (398 M^0.75^ kJ) and so 10% of that (39.8 M^0.75^ kJ) would be drawn from fat catabolism (Coltrane and Barboza 2010; Coltrane et al. 2011). We tested the sensitivity of model predictions for porcupine activity to the value of fat catabolism also using 20% of energy from fat catabolism. NEG*a* for both species can be described as

3a $${\text{NEG}}a = \, \left( {I{-}A*RMR} \right)*{\text{twhen T}}a > TLC$$

3b $${\text{NEG}}a = \, \{ I{-}A*(C*(TLC{-}{\text{ T}}a) \, + RMR)\} *{\text{twhen T}}a \le TLC$$

where *I* is the energy intake rate, *A* is an activity multiplier (the factor by which energy expenditure is increased from inactivity to activity), *RMR* is the resting metabolic rate in the thermal neutral zone (in kJ/h), *C* is the thermal conductance (in kJ h^−1^ °C^−1^), T*a* is the ambient temperature (^o^C), *TLC* is the lower critical temperature (^o^C), and t is the activity time (in h) (Humphries and Umbanhowar 2007; Studd et al. 2020). We calculated parameters *I* and *A* not for a specific behavioral category (e.g., foraging) but for the collective active state (Methods S3), to allow the comparison of predicted to observed activity time on a daily scale. Given that energy intake likely varies according to resource quality and availability, we varied the value of *I* by 10% for both species to examine the sensitivity of model predictions and then selected the *I* value that minimized residuals of predicted values from observed values (Methods S3). Energy intake of hares can also vary through time, especially for cyclic populations (Krebs et al. 2018). However, the hare populations we studied (Wisconsin, USA) and where we obtained the value of *I* from (Idaho, USA) were non-cyclic (Keith 1990; Murray et al. 2002) and thus were likely free from this additional consideration. Thermoregulatory costs calculated as the product of *C* and the difference between *TLC* and T*a* are based on the Scholander–Irving model (Scholander et al. 1950).

EE*i* is represented as

4a $${\text{EE}}i = RMR*\left( {{24 } - {\text{ t}}} \right) \quad {\text{when T}}a > TLC$$

4b $${\text{EE}}i = \, \{ \left( {C{-}C*Q} \right)*(TLC - {\text{ T}}a) \, + RMR\} *\left( {{24 }{-}{\text{ t}}} \right){\text{when T}}a \le TLC$$

where *Q* is a quality of thermal refuge that ranges from 0 to 1, with 0 representing no mitigation of thermoregulatory costs by the thermal refuge and 1 representing a perfect elimination of thermoregulatory costs by the thermal refuge (Humphries and Umbanhowar 2007; Studd et al. 2020). *Q* was set to non-zero value for porcupines because they spend a large amount of time resting in their dens in winter (Mabille et al. 2011), while *Q* was set to 0 for hares because they do not utilize thermal refuge (see Methods S3 for details). For both species, using the data on mean daily temperature and body mass of each individual (at the time of collar deployment), we used the models to predict daily activity time (h) for each individual for each day. Finally, we predicted daily NEG for each individual for each day, using the models with the values of mean daily temperature and observed daily activity time and body mass of each individual.

### Statistical analysis

To examine how different individuals behaviorally respond to the same environmental conditions, we quantified the degree of consistency of activity among different individuals as the Pearson correlation coefficient (*r*) of daily activity time on the same day between two individuals. We compared all the possible pairs of two individuals monitored for each day (e.g., for a day on which 4 individuals were monitored, there would be ${C}_{2}^{4}$ = 6 pairs). We considered that a strong positive correlation represents a high degree of consistency of activity within the population, implying that different individuals respond uniformly to the same environmental conditions.

To investigate the mechanism underlying the energy-safety trade-off in activity, we examined what environmental factors affect the deviation of observed activity time from the activity time predicted by our energetics-based models (i.e., the deviation from maintenance of energy balance). Deviation of activity time (h), defined as the subtraction of predicted from observed daily activity time for each individual for each day, was analyzed as the response variable in a linear mixed effects model (LMM) framework. We tested the effects of wind speed, snow depth and lunar luminosity (fraction of the moon illuminated combined with cloud coverage; see Methods S1 for details) while accounting for the potential effects of overwinter changes in night length and resource availability as time of winter (number of days since December 1st) as well as the effect of sex of the individual. We considered lunar luminosity as a predation risk factor (Griffin et al. 2005), and snow depth being associated with both energetics (through its effect on locomotory costs; e.g., Martin et al. 2020) and predation risk (through its effect on prey escape ability; e.g., Sullender et al. 2023). We considered wind speed as a predation risk factor, based on previous findings that the negative effect of wind speed on activity is temperature-independent in hares (Studd et al. 2022) and that RMR does not significantly change with an increase in wind speed even below the T*LC* in porcupines (DeMatteo and Harlow 1997). Time of winter and sex were included as an interaction term because males increase the level of activity in late winter for mating (Ellsworth et al. 2016) and most female porcupines are pregnant during the winter every year (Roze 2009). In addition to these explanatory variables as fixed effects, we also considered individual animal ID as random intercept to examine potential inter-individual variation in the deviation of activity from maintaining energy balance. First, we fit the model to the entire dataset for each species by additionally including ambient temperature as a binary fixed effect (0 = T*a* > T*LC*, 1 = T*a* ≤ T*LC*), considering that our energetic-based models predicted activity time of animals differently for below and within the thermoneutral zone (TNZ) (Eqs. 3 and 4). This binary variable of temperature explained the large amount of variation in the activity deviations (Table S3–4), as we expected. Therefore, we focused on the cases where the ambient temperature was above the species-specific T*LC* in the deviation analysis, to reveal the effects of environmental factors on energy-safety trade-off in activity when the effect of temperature is negligible. We standardized wind speed, snow depth, and lunar luminosity by mean-centering, and then dividing by their standard deviations, to compare the effect sizes. In addition, we used the estimated coefficients of the environmental factors in the original scales and our energetic-based model, to translate predicted changes in activity time (h) induced by the environmental factors into changes in the daily energy budget (kJ). We confirmed that none of the numeric explanatory variables exhibited |r|≥ 0.7 for each species, implying lack of effects of collinearity on model estimation (Dormann et al. 2013). We also verified that including individual ID as a random intercept improved model fit for both species (Table S5) based on the corrected Akaike information criterion for small sample size (AICc) (Burnham and Anderson 2002). For porcupines, we additionally examined whether body mass upon capture would affect the activity deviations from model prediction and the magnitude of random intercept. We have tested this potential effect of state dependence on activity deviations only for porcupines, considering that lack of fat reserves and resulting need for constant foraging (Whittaker and Thomas 1983) can minimize individual variation in the energetic state. We constructed the models with the *lmer* function of the *lme4* package in R (Bates et al. 2015) and assessed the model assumptions including normality of residuals and homogeneity of variance (Zuur et al. 2009) using the R package *performance* (Lüdecke et al. 2021), finding no serious violations.

## Results

### Observed activity

We obtained activity data from 29 hares (11 females, 17 males and 1 unknown sex) and 21 porcupines (13 females and 8 males) by accelerometers in winters 2022–2023, which produced 800 and 365 whole-day activity data points for hares and porcupines, respectively. On average, daily activity time was 12.0 h ± 1.6 (standard deviation) and 4.6 h ± 1.4 for hares and porcupines, respectively (Figure S2a). Hares and porcupines were active for 9.2 h ± 1.4 and 2.9 h ± 1.4 at night on average, respectively (Figure S2b). Daytime activity time was 2.1 h ± 0.8 and 1.5 h ± 1.4 on average for hares and porcupines, respectively (Figure S2c).

**Fig. 1:**
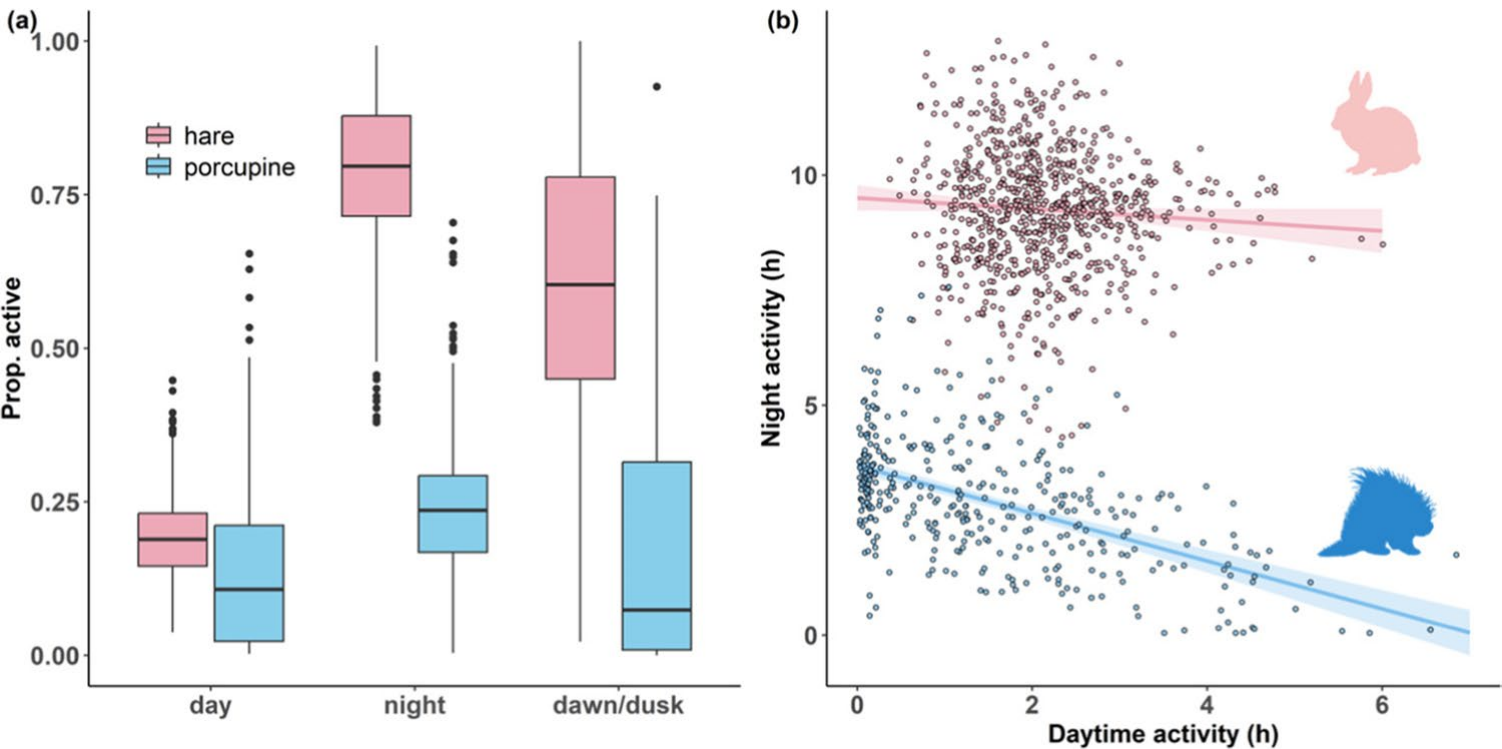
**a** Observed diel activity patterns in winter of snowshoe hares *Lepus americanus* and North American porcupines *Erethizon dorsatum* in central Wisconsin, USA shown as the proportion of time being active during each diel phase of the day, and **b** observed relationship between daytime activity and nighttime activity on the same day of hares (*r* = − 0.07) and porcupines (*r* = − 0.53) in winter shown with regression lines with 95% confidence intervals

Both species were generally nocturnal; hares and porcupines were active 78.6% and 24.1% of the time at night on average, respectively (Fig. 1a). The degree of nocturnality was higher for hares than for porcupines, with the majority of daily activity consistently occurring at night in hares. Daytime and nighttime activity time on the same day were not correlated in hares (*r* = − 0.07; Fig. 1b). However, for porcupines, there were many cases where the majority of daily activity occurred during the daytime, and daytime and nighttime activity time on the same day were negatively correlated (*r* = − 0.53; Fig. 1b). As winter proceeded, the length of night shortened causing the proportion of time being active at night of hares to approach 1 in late winter (Figure S3a). In contrast, both nighttime and daytime activity times of porcupines were consistently far below night and day length over the winter, respectively (Figure S3b).

**Fig. 2:**
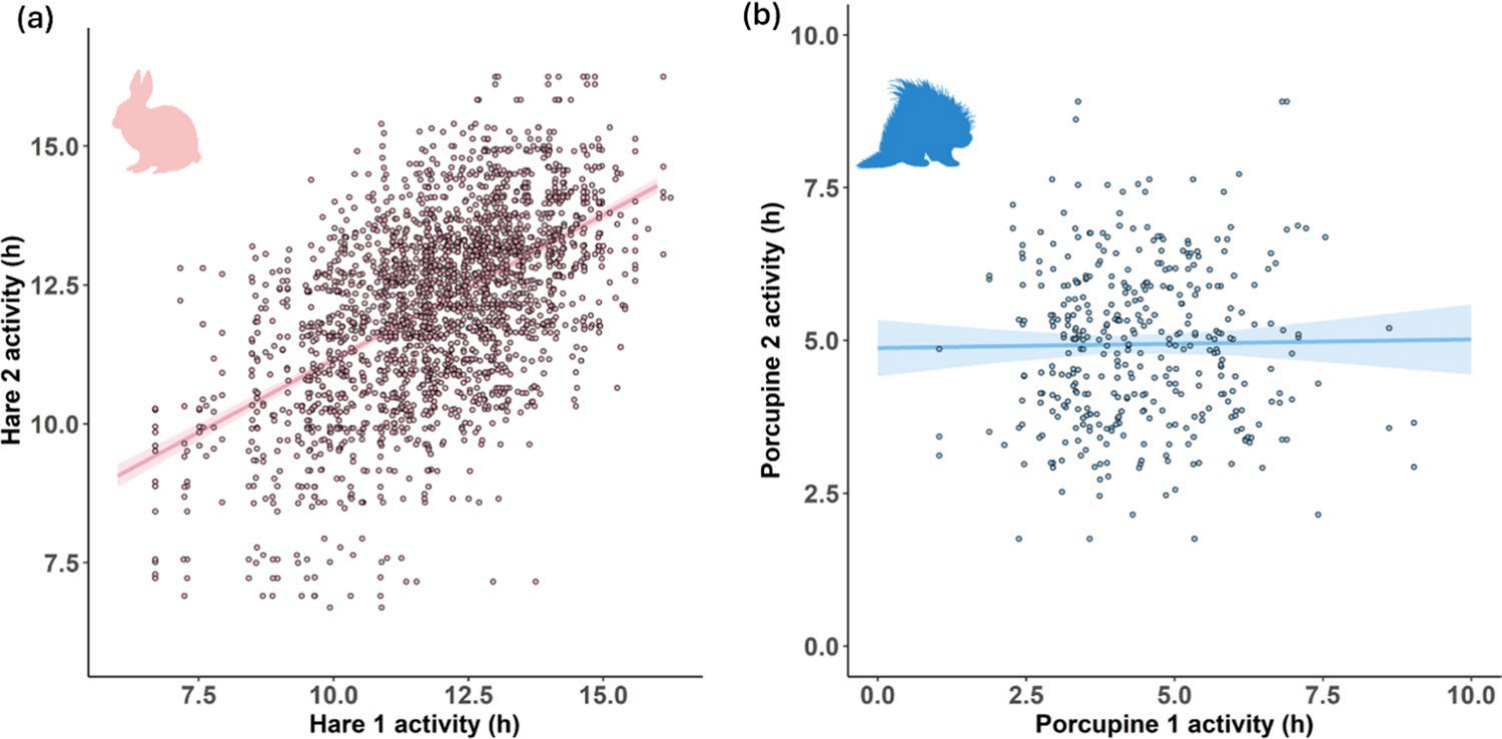
Correlation of daily activity time between two different individuals on the same day in winter **a** in snowshoe hares *Lepus americanus* (*r* = 0.55) and **b** in North American porcupines *Erethizon dorsatum* (*r* = 0.014) in central Wisconsin, USA, presented with the regression lines with 95% CIs

Correlation of daily activity time between two individuals on the same day was moderately positive for hares (*r* = 0.55; Fig. 2a), demonstrating that different individuals relatively uniformly responded to the same environmental conditions. However, we did not detect activity consistency among different individuals in porcupine (*r* = 0.014; Fig. 2b).

### Predicted activity time based on energetics

**Fig. 3:**
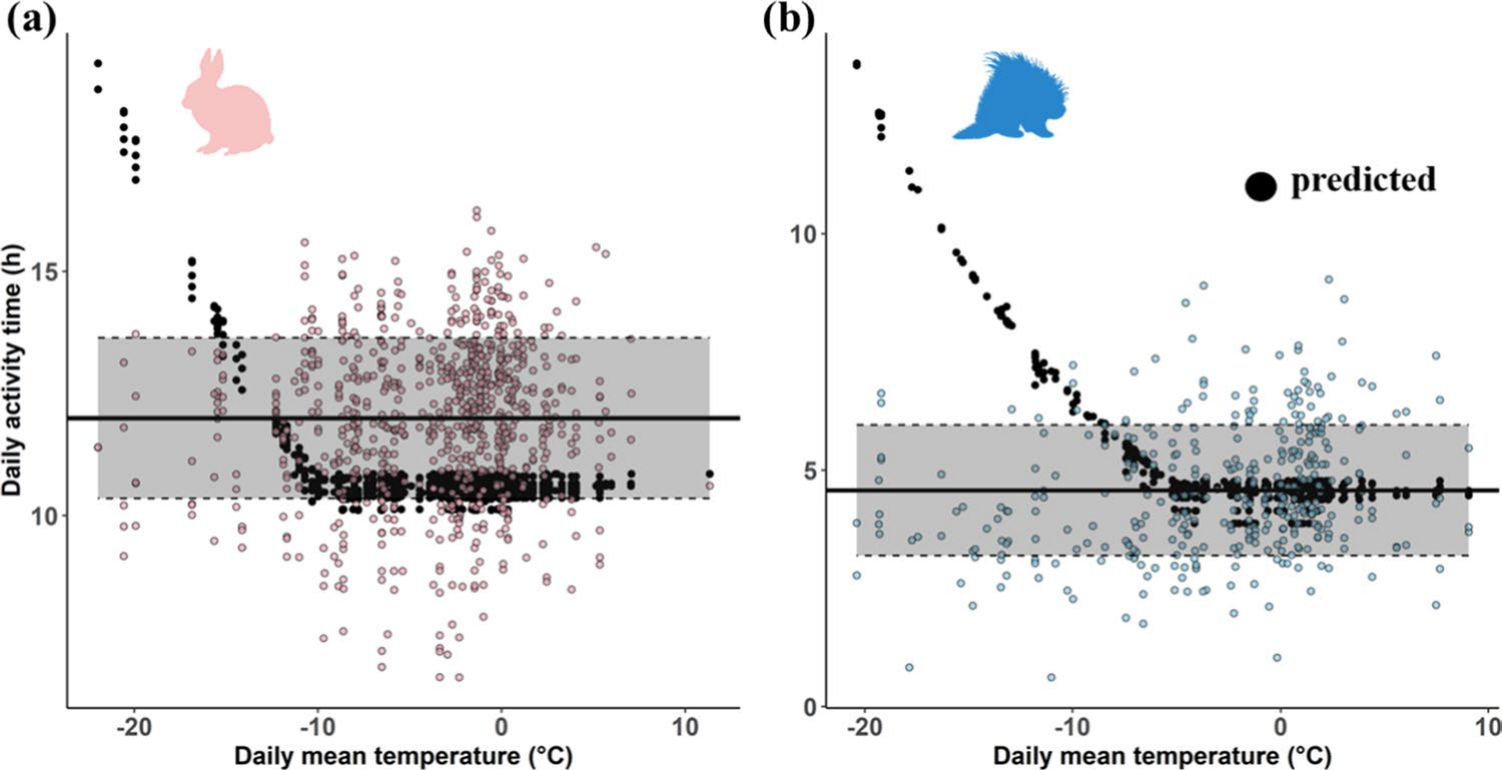
Daily activity time in winter predicted by the energetics-based activity model presented with observed activity time, for **a** snowshoe hares *Lepus americanus* and **b** North American porcupines *Erethizon dorsatum* in central Wisconsin, USA. Black points are the predicted values, points with colors are the observed values, solid horizontal lines represent the means of observed activity time, and dotted horizontal lines represent the mean ± standard deviation of the observed activity time, for each species

Our energetics-based model predicted that daily activity time would increase as ambient temperature decreases below T*LC* (i.e., below the TNZ) for both species (Fig. 3). Coefficient of variation (CV; calculated as the ratio of the root mean squared error to the mean of the observed values) based on the model was 0.199 and 0.557 for hares and porcupines, respectively.

Although our model predicted an increase in activity time with decreasing temperature below the TNZ, the observed activity time was relatively constant for both species. Specifically, observed activity time was far below the predicted values at low temperatures (Fig. 3). Increasing the value of daily energy requirement obtained from fat catabolism in porcupines resulted in lower predicted activity times, though the model still overpredicted activity time below the TNZ (Figure S5). This caused predicted daily NEG to be negative in most cases when temperature was lower than T*LC* for both species, demonstrating that hares and porcupines likely failed to maintain energy balance at low temperatures (Figure S6). Predicted values were generally within the range of the observed activity time for both species when temperatures were within the TNZ, but variation in the observed values around the predicted values was still present (CV = 0.187 for hares and CV = 0.298 for porcupines within their TNZs).

### Deviation of activity from energy balance

**Fig. 4:**
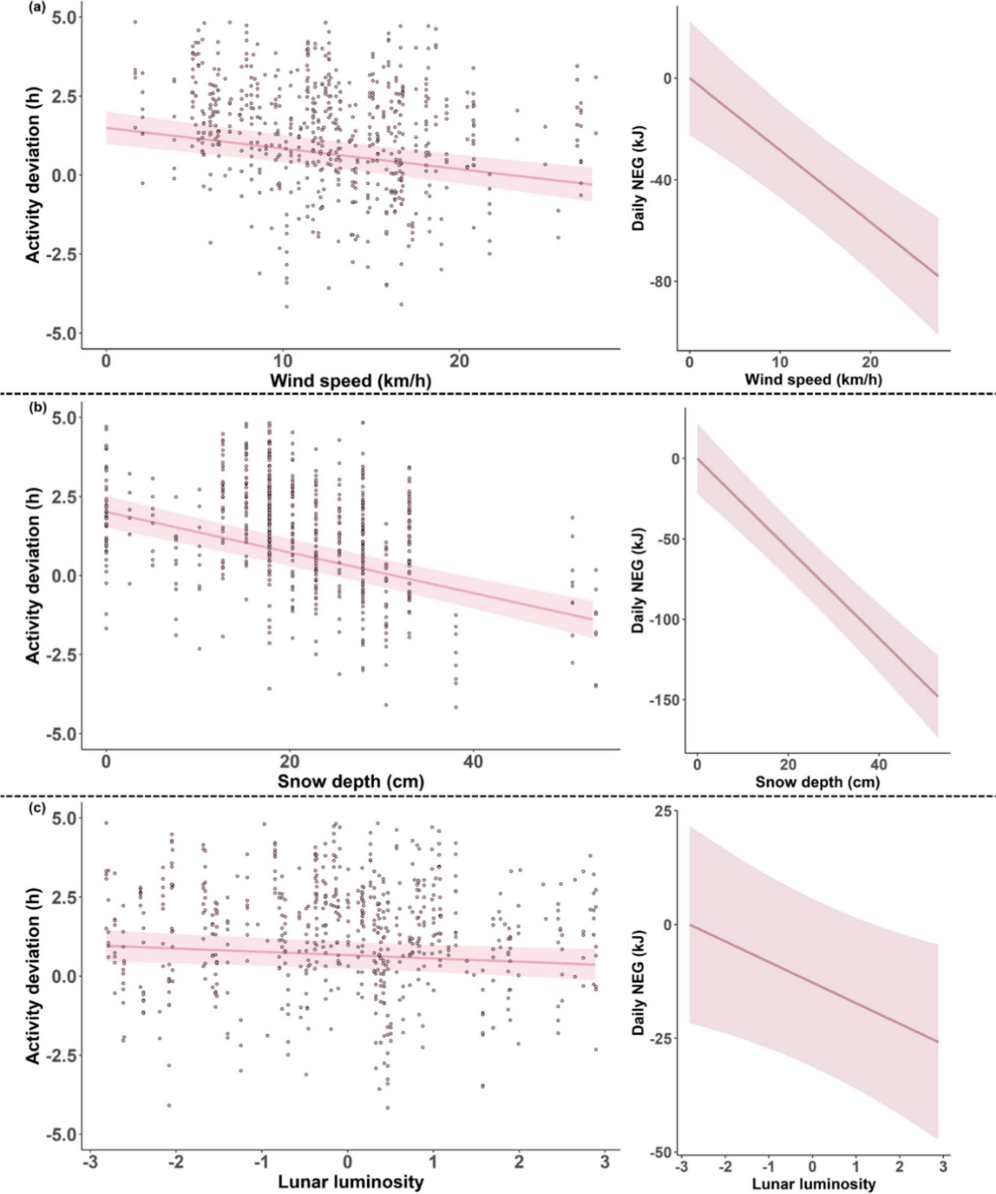
Effects of **a** daily wind speed, **b** daily snow depth, and **c** lunar luminosity on the deviations of observed daily activity time from energy balance and on the daily net energy gain (NEG) of snowshoe hares *Lepus americanus* in winter in central Wisconsin, USA, predicted by the linear mixed effects models. Predicted values are shown with 95% CIs, and small dots represent raw data points. Daily NEG was predicted for the average hare in our study site (1.35 kg) using the energetics-based model for TNZ and predicted changes in activity deviations, setting y-intercept to 0

The model predicting the deviation of the observed daily activity time of hares from energy balance for TNZ showed that wind speed, snow depth and lunar luminosity all negatively impacted the deviations of activity time from energy balance (Fig. 4a–c). Snow depth had the largest negative effect, whereas wind speed had an intermediate effect and lunar luminosity showed the smallest effect (Table S6). Our model predicted that 20 km/h of increase in wind speed or 20 cm of increase in snow depth would result in a 1.3 h decrease in daily activity time (Fig. 4a, b), which is equivalent to 12.3% of daily activity time (10.54 h) required for the average hare in our study site (1.35 kg) to maintain energy balance when T*a* > T*LC*. This amount of reduction in daily activity time would result in a 56 kJ decrease in daily NEG (Fig. 4a, b), which is equivalent to 7.4% of the daily energy expenditure of the average hare (754.3 kJ/day for TNZ). Negative deviations of hare activity time from energy balance increased as winter progressed regardless of sex (Table S6), likely due to the decrease in night length coupled with the constant amount of time being active during daytime (Figure S3). 17.6% of the variance in activity deviations of hares unexplained by the fixed effects was attributed to the random intercept (Table S6).

For porcupines, the model demonstrated that none of the environmental factors explained the deviations of activity from energy balance for TNZ (Table S7). In contrast to hares, positive deviations of porcupine activity time from energy balance increased as winter proceeded regardless of sex (Table S7). Male porcupines exhibited more positive activity deviations from energy balance than females, and 44.1% of the variance in activity deviations of porcupines unexplained by the fixed effects was attributed to the random intercept (Table S7). These patterns remained unchanged when the value of daily energy requirement obtained from fat catabolism was increased (Table S8). In addition, including body mass neither improved model fit nor affected the relative importance of the random intercept (Table S9).

## Discussion

The observed daily activity time of both hares and porcupines were notably lower than predicted by the energetics-based model when temperatures were below the TNZ. This demonstrates that both species attempted to minimize energy expenditure at low temperatures via an energy conservation mode rather than actively acquiring energy. For hares, which we found to be more strictly nocturnal than porcupines, night length seemingly constrained their activity time. Further, the deviation of observed activity time from that predicted just for energy balance when temperatures were within the TNZ appeared to be driven by different mechanisms in hares and porcupines, highlighting divergent patterns of energy-safety trade-off between them. Specifically, hare activity was governed by multiple environmental factors associated with predation risk and potentially energetics. In contrast, no environmental factors appeared to drive deviations of porcupine activity from energy balance. Furthermore, we observed more individual variation in activity deviation from energy balance and inconsistent activity patterns among porcupines. In contrast, hares exhibited limited individual variation in activity deviations from energy balance and relatively uniform responses in their activity to environmental conditions.

While our model assumed that prey animals minimize daily activity time while maintaining energy balance (Schoener 1971), it overpredicted activity for both species when temperatures were below the TNZ. The model predicted that animals could compensate for increased thermoregulatory costs under low temperatures by increasing activity (foraging) time, but both species maintained the same or slightly lower level of activity time as when temperatures were within the TNZ. Reduction of activity with decreasing temperature in winter is not uncommon among mammalian prey (Orrock and Danielson 2009). The reason why hares and porcupines did not increase activity for energy compensation may be due to predation risk associated with activity; prey theoretically should terminate foraging once the benefits (energy gain) stop exceeding the costs including predation risk (Brown and Kotler 2004). In addition, when resource availability is low, energetic benefits of activity including foraging become limited under low temperatures, leading to a reduced level of activity (Humphries et al. 2005). These may be more relevant to porcupines, considering that they possess fat reserves (Coltrane et al. 2011) and resting in protected natural shelters provides benefit in risk avoidance (Kowalczyk and Zalewski 2011) in addition to the thermoregulatory benefit. It was also reported that risk of predation from Canada lynx (*Lynx canadensis*) was higher for hares at lower temperatures despite lower activity level of hares (Peers et al. 2020), implying that predation risk can be higher under low temperatures possibly due to increasing energetic demands of predators.

Previous studies have reported that nocturnal mammals increase diurnal activity when energetically stressed (van der Vinne et al. 2019; Guiden and Orrock 2020). This appears to be the case for porcupines, considering that they are less strictly nocturnal and their nighttime activity time is negatively correlated with daytime activity time on the same day. In contrast, shifting the timing of activity was impossible for hares, given their strict nocturnality. Indeed, we did not detect a correlation between nighttime and daytime activity time on the same day in hares. In addition, hares maintained nighttime activity time throughout the winter despite shortening night length, while their proportion of time being active during daytime stayed constant despite the overwinter increase in day length. This inflexibility for the timing of activity of hares likely led to overwinter increase in expected daytime energy deficit, considering that hare activity increasingly deviated in a negative way from energy balance from early to late winter. This finding demonstrates that day/night length can have divergent effects on animal activity and resulting energetics depending on the flexibility of diel activity patterns.

Although the effects of species traits on antipredator responses are increasingly studied (Wirsing et al. 2021), how prey traits mediate their activity by affecting the balance between energy acquisition and risk avoidance remains relatively underexplored. For hares, deviations of observed activity time from energy balance were driven by multiple environmental factors that relate best to predation risk. As expected (Studd et al. 2022), hares were less active than expected based on maintenance of energy balance with increasing wind speed. Snow depth had the largest negative effect on activity deviations of hares, which is consistent with previous findings (Peers et al. 2020). This is likely through either energetic (lower food intake rate; Robinson and Merrill 2012, or increased locomotory costs; Parker et al. 1984) or risk pathway (impaired escape ability from predators; Sullender et al. 2023), or both. The activity responses of hares we detected highlight the energy-safety trade-off they are facing; our models predicted that 20 km/h of increase in wind speed or 20 cm of increase in snow depth would result in 12% of reduction in daily activity time required for maintaining energy balance, leading to an energy deficit of 7% that day. Lunar luminosity had the smallest, but still significant negative effect on activity deviations of hares, which should be due to heightened predation risk (Griffin et al. 2005). Contrary to hares, none of the environmental factors explained the deviation of observed activity from energy balance for porcupines. This is likely because porcupines are morphologically protected and, thus, less dependent on behavioral risk avoidance. We expected that snow depth would negatively impact porcupine activity through increased locomotory costs in addition to through heightened predation risk. However, locomotory costs generally account for a small proportion of energetic costs of activity (Karasov 1992), and this trend may be pronounced for semi-arboreal species such as porcupines that do not spend much time traveling on the ground. Overall, these results demonstrate that porcupines may not trade-off energy gain through activity for safety as hares do, highlighting that prey traits potentially play a key role in the mechanism underlying prey activity through mediating the energy-safety trade-off.

Prey encounter rate is the key component of hunting success of predators (Lima and Dill 1990), and predator–prey encounter rate is a function of predator and prey activity (Hutchinson and Waser 2007; Pawar et al. 2012). Predators are thus likely to match their activity pattern with that of their prey (Lang et al. 2019). Our results reveal that different individual hares showed similar activity patterns under the same environmental conditions and limited variation in activity deviations from energy balance. In contrast, activity patterns were inconsistent among individuals in porcupines, and their activity was largely independent of environmental factors. In addition, porcupines showed more individual variation in activity deviations from energy balance compared to hares. The balance between energy acquisition and risk avoidance is often mediated by the energetic state of an individual (McNamara and Houston 1990; Bednekoff and Houston 1994), which can explain intra-specific individual variation in activity patterns. However, body mass of porcupines did not affect their activity deviations from energy balance. These results suggest that relaxed energy-safety trade-off induces behavioral flexibility in porcupines. Morphological defense appears to enable porcupines to maintain their activity level under high predation risk (McLean and Godin 1989, Abrams 1995). Furthermore, possession of fat reserves by porcupines may facilitate flexibility in making the decision whether to be active or not under unfavorable biotic and abiotic conditions by mitigating foraging needs. In contrast, prey that lack these morphological defense and/or energy reserve, like hares, should have less behavioral flexibility due to tightened energy-safety trade-off and thus be forced to be active at predictable time or intervals. Consequently, combined with the different degrees of flexibility in nocturnality mentioned earlier, it can be easier for predators to match their activity patterns with activity patterns of hares likely leading to higher predator–hare encounter and predation rates. Our findings may help to explain the high predation rate observed in hares (Krebs et al. 2018) compared to porcupines (Mabille et al. 2010), providing a potential explanation for the different levels of top-down regulation of prey populations inhabiting the same ecological communities. Specifically, prey that lack morphological protection and exhibit predictable activity patterns, like hares, appear to be a more reliable food source of predators and may contribute to their role as keystone prey (Mills et al. 1993). Overall, our findings highlight that species traits play an important role in structuring activity patterns of prey through mediating energy-safety trade-off and thereby potentially governing the demographic patterns and ecological roles of prey.

## Supplementary Information

Below is the link to the electronic supplementary material.

Supplementary file1 (DOCX 1556 KB)

## Data Availability

Data available at figshare 10.6084/m9.figshare.30132247.

## Code availability

Code available at figshare 10.6084/m9.figshare.30132247.
