# Supplementary material for: Energy-safety trade-offs differ between winter-adapted species of prey to drive distinct activity patterns
Source: Oecologia. 2025 Sep 22;207(10):162. doi: 10.1007/s00442-025-05797-y (PMC12454541; doi:10.1007/s00442-025-05797-y)
Supplement: Supplementary file 1 — Supplementary file1 (DOCX 1556 KB) [file 442_2025_5797_MOESM1_ESM.docx]

**Supplementary Materials**

**Methods S1-Meteorological and environmental data**

We obtained meteorological data including wind speed, cloud coverage (categorical measure; clear = 0 % cloud coverage, few = 0 < % ≤ 25, scattered = 25 < % ≤ 50, broken = 50 < % < 100 and overcast = 100 %) and air temperature recorded every 20-60 minutes as a part of the Automated Surface Observing Systems (ASOS) at the nearest available weather station to the study areas: for hares, Taylor County Airport (45.101° N, 90.303° W); for porcupines South Wood County Airport (44.359° N, 89.837° W). Both weather stations are located ~ 30 km away from their corresponding study site. We also obtained the data on daily snow depth from the nearest available weather station to each study area: Medford USC00475255 (45.131° N, 90.344° W) for hares; Mather 3NW USC00475164 (44.167° N, 90.333° W) for porcupines, both of which are located ~ 25 km away from their corresponding study sites.

We additionally calculated the fraction of the moon illuminated by the *getMoonIllumination* function of the *suncalc* package in R (Thieurmel and Elmarhraoui 2022), and then derived the index of the level of lunar luminosity from the fraction of the moon illuminated and cloud coverage. We first converted the cloud coverage data from categorical to numeric variables (clear = 0, few/scattered = 1, broken = 2 and overcast =3), and calculated the average cloud cover value for each night (ranged from 0-3). We then subtracted the scaled (i.e., mean-centered and then divided by its standard deviation) cloud cover value from the scaled fraction of the moon illuminated in our data frame.

**Methods S2-Behavioral classification of porcupine accelerometer data**

We recorded time-stamped behaviors of collared porcupines with a digital video camera either upon release or by locating them using VHF. We recorded a total of 15.32 h of video from the 21 collared porcupines, and porcupine behavior in the videos was classified into four categories: foraging (feeding with a small movement), climbing/descending (traveling on/in a tree), traveling on the ground, and resting. We then assigned observed behaviors to corresponding accelerometer data for subsequent behavioral classification. To deal with a potential small time-inconsistency between the videos and accelerometers, we examined a difference in time between each video and accelerometer by identifying occasions in which a porcupine transitioned from resting to climbing/descending or traveling on the ground and then made a time-adjustment if needed (Studd et al. 2019). To classify accelerometer data from porcupines, we trained Random Forest algorithm using AcceleRater, an open-access web application developed by Resheff et al. (2014).

We used the accelerometer data with observed behaviors assigned (15.32 h in total) summarized in 10-second time windows as the training dataset, computing all the available 52 summary statistics (default setting in AcceleRater) and assessing accuracy, precision and recall by five-fold cross-validation. We first trained the algorithm only for classifying behavior into active state (foraging, climbing/descending and traveling on the ground) and inactive state (resting), and Random Forest algorithm classified an active state with an accuracy of 95.0 % (Table S1). We then trained the algorithm for further classifying an active state (2.86 h of training dataset) into specific behavioral categories, and Random Forest was shown to classify foraging, climbing/descending and traveling on the ground with accuracies of 88.8 %, 86.7 % and 94.9 %, respectively (Table S1). Finally, by using the trained Random Forest algorithm, we annotated the full porcupine accelerometer dataset (summarized in 10-second time windows) including data points without observed behavioral assignment.

**Methods S3-Energetics-based activity model**

Resting metabolic rate (RMR) of hares (976.68 ml O_2_/h; Sheriff et al. 2009) and porcupines (230.00 ml O_2_/kg/h; DeMatteo & Harlow 1997)) were converted to kJ/h by multiplying by 0.0201kJ/ml O_2_ (Montevecchi et al. 1992) and then scaled with body mass using the scaling factor 0.75 (Savage et al. 2004) and the average body mass of the animals reported in the studies from which we obtained the original RMR values (hares: 1.4 kg from Sheriff et al. 2009, porcupines: 10.7 kg from DeMatteo and Harlow 1997).

Thermal conductance (*C*) of hares (30.90 ml O_2_/h/°C; Sheriff et al. 2009)) and porcupines (0.012 ml O_2_/g/h/°C; Fournier and Thomas 1999) were also converted to kJ/h by multiplying by 0.0201 and then scaled with body mass using the scaling factor 0.57 (Riek and Geiser 2013, Fristoe et al. 2015) and the average body mass of the animals reported in the studies from which we obtained the original RMR values (hares: 1.4 kg from Sheriff et al. 2009, porcupines: 6.1 kg from Fournier and Thomas 1999).

Lower critical temperature (T*LC*) of hares (-10 °C) was obtained from Sheriff et al. (2009). For porcupines, since the reported values were not consistent among the studies (from -2 °C in Fournier and Thomas 1999 to -12 °C in Irving et al. 1955), we assumed that their T*LC* in our study area is -5 °C.

To obtain activity multiplier (*A*), we followed (Karasov 1992) to estimate activity-related metabolic rate (AMR; metabolic rate during activity) based on field metabolic rate (FMR, which is the sum of AMR and RMR), proportion of time being active over 24 h and RMR reported in previous studies for both species. For hares, it has been reported that FMR of free-ranging hares in winter is 808.78 kJ/day (reported as 742 kJ/kg/day from hares with the average body mass of 1.09 kg in Ellsworth et al. 2016) while RMR is 19.63 kJ/h (reported as 976.68 ml O_2_/h in Sheriff et al. 2009), and that free-ranging hares are active 48.6 % of a day on average in winter (Ellsworth et al. 2016). Based on these values, AMR was estimated to be 48.58 kJ/h and thus *A* = 48.58 / 19.63 = 2.47. For porcupines, FMR of free-ranging individuals in winter has been estimated to be 1881.49 kJ/day (reported as 440 kJ/kg^0.77^/day from porcupines with the average body mass of 6.6 kg in Coltrane et al. 2011) while RMR is 49.47 kJ/h (reported as 230.00 ml O_2_/kg/h from porcupines with the average body mass of 10.7 kg in DeMatteo and Harlow 1997). Additionally, Coltrane and Sinnot (2013) reported that free-ranging porcupines are active 25 % of a day on average in winter. Based on these values, AMR was estimated to be 165.17 kJ/h and thus *A* = 165.17 / 49.47 = 3.34. Both values of *A* are consistent with analogous calculations in other terrestrial mammals (Karasov 1992)

Ellsworth et al. (2016) reported FMR (808.78 kJ/day) and daily activity rate (0.486) of free-ranging hares in winter, and the hares they monitored did not lose body mass over the winter, suggesting that they were maintaining energy balance. Based on these findings, default energy intake rate (*I*) of free-ranging hares in winter was estimated to be (808.78 kJ) / (24h*0.486) = 69.34 kJ/h, and this mass-free *I* was scaled with body mass based on the average winter body mass of the hares (1.09 kg) reported in Ellsworth et al. (2016) and the scaling factor 0.64 for hindgut fermenters (Clauss et al. 2007). Porcupines, in contrast, gain 411 kJ/day from fat catabolism on average in winter (Coltrane and Barboza 2010, Coltrane al. 2011). Provided their daily activity time (24*0.25 = 6 h, Coltrane and Sinnot 2013), FMR (49.47 kJ/h, DeMatteo and Harlow 1997) and *A* (3.34), based on the equations (3a) and (5a) in the main text,

(I - 3.34*49.47 kJ/h)*(6 h) - 49.47 kJ/h*(24 h - 6 h) = -411 kJ/day

Solving this equation gives *I* = 245.14 kJ/h for free-ranging porcupines in winter, and this mass-free *I* was scaled with body mass based on the average winter body mass of the porcupines (6.6 kg) reported in Coltrane et al. (2011) and the scaling factor 0.64 for hindgut fermenters (Clauss et al. 2007). Since energy intake rate is potentially variable among different geographical regions due to differences in resource quality or availability, we estimated the predicted daily activity time by increasing and decreasing the default energy intake rate for both species by 10 % to examine the sensitivity of model prediction to the value of energy intake rate. We then adopted the most plausible intake rate (among the default value and 90 % and 110 % of the default value), which produced the smallest root mean square deviation for above T*LC*, for the subsequent analyses. For both species, 90 % of the default intake rate was shown to be the most plausible value (Figure S4).

Refuge quality (*Q*), which ranges from 0 to 1, represents the degree to which thermal refuge (e.g., nest or den) mitigates thermoregulatory costs while an animal is resting when T*a* ≤ T*LC* (Humphries and Umbanhowar 2007, Studd et al. 2020). *Q* = 0 represents no mitigation of thermoregulatory costs by the thermal refuge, while *Q* = 1 represents a perfect elimination of thermoregulatory costs by the thermal refuge (Humphries and Umbanhowar 2007, Studd et al. 2020). We only considered *Q* in the model for porcupines, provided that porcupines spend a large amount of time resting in their dens in winter (Mabille et al. 2011) whereas hares do not utilize thermal refuge. Mabille et al (2011) reported that power consumed by porcupine mounts was around 25 % lower in dens than outside dens when T*a* ≤ T*LC* in winter, and thus we assumed that *Q* = 0.25. See Table S2 for the list of all the parameter values.

| Table S1. Recall, precision and accuracy values obtained for active and inactive phases (upper) and for three behavioral categories of active phase (lower) by the Random Forest algorithm trained for classifying porcupine accelerometry data. See Resheff et al. (2014) for the definition of each term. | | | | |
| --- | --- | --- | --- | --- |
|  | **Active** | **Inactive** | **Weighted average** |  |
| **Recall** | 86.58 | 96.88 | 94.96 |  |
| **Precision** | 86.41 | 96.92 | 94.96 |  |
| **Accuracy** | 94.06 | 94.96 | 94.96 |  |
|  | **Climb/descend** | **Forage** | **Travel on ground** | **Weighted average** |
| **Recall** | 76.76 | 93.65 | 76.19 | 85.21 |
| **Precision** | 81.82 | 85.59 | 91.43 | 85.30 |
| **Accuracy** | 86.67 | 88.81 | 94.94 | 89.11 |

| Table S2. List of the parameters used in or relevant to our energetics-based activity model for snowshoe hares and porcupines, with reference from which parameter values were obtained. Parameter values used in predicting activity time by the model are shown in bold. SF represents scaling factor. | | | | |
| --- | --- | --- | --- | --- |
|  | Hare | Porcupine | Hare reference | Porcupine reference |
| Resting metabolic rate (*RMR*) | 976.68 ml O_2_/h | 230 ml O_2_/kg/h | Sheriff et al. 2009 | DeMatteo and Harlow 1997 |
| **converted *RMR*** | **15.25 kJ/kg^0.75^/h** | **8.36 kJ/kg^0.75^/h** | SF = 0.75 from Savage et al. 2004 | |
| Thermal conductance  (*C*) | 30.90 ml O_2_/h/°C | 0.012 ml O_2_/g/h/°C | Sheriff et al. 2009 | Fournier and Thomas 1999 |
| **converted *C*** | **0.51 kJ/kg^0.57^/h/°C** | **0.52 kJ/kg^0.57^/h/°C** | SF = 0.57 from Riek and Geiser 2013 and   Fristoe et al. 2015 | |
| **Lower critical temperature  (T*LC*)** | **-10 °C** | **-5 °C** | Sheriff et al. 2009 | Fournier and Thomas 1999 Irving et al 1955 |
| **Activity multiplier (*A*)** | **2.47** | **3.34** | Calculated**^1^** | Calculated**^1^** |
| Energy intake rate (*I*) | 69.34 kJ/h | 245.14 kJ/h | Calculated**^1^** | Calculated**^1^** |
| **converted *I*** | **65.62 kJ/kg^0.64^/h** | **73.27 kJ/kg^0.64^/h** | SF = 0.64 from Clauss et al. 2007 | |
| **Refuge quality (*Q*)** | **NA** | **0.25** | NA | Mabille et al. 2011 |

**^1^Calculated using values from multiple sources, as described in the text**

| Table S3. Parameter estimates from the linear mixed effects model explaining the deviations of observed activity time of snowshoe hares *Lepus americanus* from the prediction by the energetics-based model. Wind is the mean daily wind speed, snow is the daily snow depth, luminosity is lunar luminosity (fraction of the moon illuminated combined with cloud coverage), winter is time of winter (number of days since December 1st), and TNZ is a binary variable (0 = T*a* > T*LC*, 1 = T*a* ≤ T*LC*). Wind, snow and luminosity are scaled by mean-centering and then being divided by their standard deviations. | | | | | |
| --- | --- | --- | --- | --- | --- |
| **Fixed effect** | **Estimate** | **SE** | **t value** | **p-value** | |
| Intercept | 2.29 | 0.50 | 4.58 | <0.001 | |
| Wind (scaled) | -0.47 | 0.07 | -7.01 | <0.001 | |
| Snow (scaled) | -0.69 | 0.08 | -9.12 | <0.001 | |
| Luminosity (scaled) | -0.19 | 0.07 | -2.60 | 0.009 | |
| Winter | -0.02 | 0.01 | -3.35 | <0.001 | |
| Sex: male | 0.46 | 0.64 | 0.73 | 0.47 | |
| Winter*Sex: M | 0.01 | 0.01 | 0.84 | 0.40 | |
| TNZ: T*a* ≤ T*LC* | -2.25 | 0.20 | -11.25 | <0.001 | |
| **Random effect** | **Variance** | **SD** | | |  |
| Hare ID | 0.4316 | 0.6569 | | |  |
| Residual | 2.5214 | 1.5879 | | |  |
| **Conditional R^2^** | 0.46 |  | | |  |
| **Marginal R^2^** | 0.37 |  | | |  |

| Table S4. Parameter estimates from the linear mixed effects model explaining the deviations of observed activity time of North American porcupines *Erethizon dorsatum* from the prediction by the energetics-based model. Wind is the mean daily wind speed, snow is the daily snow depth, luminosity is lunar luminosity (fraction of the moon illuminated combined with cloud coverage), winter is time of winter (number of days since December 1st), and TNZ is a binary variable (0 = T*a* > T*LC*, 1 = T*a* ≤ T*LC*). Wind, snow and luminosity are scaled by mean-centering and then being divided by their standard deviations. | | | | | |
| --- | --- | --- | --- | --- | --- |
| **Fixed effect** | **Estimate** | **SE** | **t value** | **p-value** | |
| Intercept | -3.13 | 0.69 | -4.52 | <0.001 | |
| Wind (scaled) | 0.12 | 0.09 | 1.37 | 0.17 | |
| Snow (scaled) | -0.14 | 0.11 | -1.28 | 0.20 | |
| Luminosity (scaled) | -0.28 | 0.10 | -2.70 | 0.007 | |
| Winter | 0.03 | 0.01 | 4.85 | <0.001 | |
| Sex: male | 1.57 | 1.32 | 1.19 | 0.24 | |
| Winter*Sex: M | -0.01 | 0.02 | -0.55 | 0.58 | |
| TNZ: T*a* ≤ T*LC* | -2.08 | 0.23 | -9.19 | <0.001 | |
| **Random effect** | **Variance** | **SD** | | |  |
| Porcupine ID | 1.236 | 1.112 | | |  |
| Residual | 2.466 | 1.570 | | |  |
| **Conditional R^2^** | 0.60 |  | | |  |
| **Marginal R^2^** | 0.40 |  | | |  |

| Table S5. Comparison of models with and without individual animal ID as a random intercept,  explaining the deviations of observed activity time of Snowshoe hares *Lepus americanus* and North American porcupines *Erethizon dorsatum* from the prediction by the energetics-based model for T*a* > T*LC*. Wind is the mean daily wind speed, snow is the daily snow depth, luminosity is lunar luminosity (fraction of the moon illuminated combined with cloud coverage), and winter is time of winter (number of days since December 1st). Wind, snow and luminosity are scaled by mean-centering and then being divided by their standard deviations. *df* is degree of freedom, and AICc is Akaike information criterion for small sample size. | | | |
| --- | --- | --- | --- |
| **Hare model** | ***df*** | **∆AICc** | **AICc weight** |
| wind + snow + luminosity + winter*sex + (1\|ID) | 9 | 0 | 1 |
| wind + snow + luminosity + winter*sex | 8 | 48.63 | 0 |
| **Porcupine model** | ***df*** | **∆AICc** | **AICc weight** |
| wind + snow + luminosity + winter*sex + (1\|ID) | 9 | 0 | 1 |
| wind + snow + luminosity + winter*sex | 8 | 78.03 | 0 |

| Table S6. Parameter estimates from the linear mixed effects model explaining the deviations of observed activity time of snowshoe hares *Lepus americanus* from the prediction by the energetics-based model for T*a* > T*LC*. Wind is the mean daily wind speed, snow is the daily snow depth, luminosity is lunar luminosity (fraction of the moon illuminated combined with cloud coverage), and winter is time of winter (number of days since December 1st). Wind, snow and luminosity are scaled by mean-centering and then being divided by their standard deviations. | | | | |
| --- | --- | --- | --- | --- |
| **Fixed effect** | **Estimate** | **SE** | **t value** | **p-value** |
| Intercept | 2.26 | 0.45 | 4.98 | <0.001 |
| Wind (scaled) | -0.34 | 0.06 | -5.77 | <0.001 |
| Snow (scaled) | -0.68 | 0.07 | -10.01 | <0.001 |
| Luminosity (scaled) | -0.14 | 0.06 | -2.29 | 0.023 |
| Winter | -0.02 | 0 | -3.54 | <0.001 |
| Sex: male | 0.19 | 0.58 | 0.32 | 0.75 |
| Winter*Sex: M | 0.01 | 0.01 | 1.33 | 0.18 |
| **Random effect** | **Variance** | **SD** |  |  |
| Hare ID | 0.3773 | 0.6142 |  |  |
| Residual | 1.7687 | 1.3299 |  |  |
| **Conditional R^2^** | 0.43 |  |  |  |
| **Marginal R^2^** | 0.31 |  |  |  |

| Table S7. Parameter estimates from the linear mixed effects model explaining the deviations of observed activity time of North American porcupines *Erethizon dorsatum* from the prediction by the energetics-based model for T*a* > T*LC*. Wind is the mean daily wind speed, snow is the daily snow depth, luminosity is lunar luminosity (fraction of the moon illuminated combined with cloud coverage), and winter is time of winter (number of days since December 1st). Wind, snow and luminosity are scaled by mean-centering and then being divided by their standard deviations. | | | | |
| --- | --- | --- | --- | --- |
| **Fixed effect** | **Estimate** | **SE** | **t value** | **p-value** |
| Intercept | -2.33 | 0.58 | -4.04 | <0.001 |
| Wind (scaled) | -0.03 | 0.07 | -0.47 | 0.64 |
| Snow (scaled) | -0.02 | 0.08 | -0.20 | 0.84 |
| Luminosity (scaled) | -0.03 | 0.08 | -0.43 | 0.67 |
| Winter | 0.03 | 0.01 | 4.17 | <0.001 |
| Sex: male | 2.70 | 1.08 | 2.49 | 0.018 |
| Winter*Sex: M | -0.02 | 0.01 | -1.63 | 0.11 |
| **Random effect** | **Variance** | **SD** |  |  |
| Porcupine ID | 0.7476 | 0.8647 |  |  |
| Residual | 0.9489 | 0.9741 |  |  |
| **Conditional R^2^** | 0.55 |  |  |  |
| **Marginal R^2^** | 0.19 |  |  |  |

| Table S8. Parameter estimates from the linear mixed effects model explaining the deviations of observed activity time of North American porcupines *Erethizon dorsatum* from the prediction by the energetics-based model for T*a* > T*LC* assuming that 20 % of their daily energy requirement would be drawn from fat catabolism. Wind is the mean daily wind speed, snow is the daily snow depth, luminosity is lunar luminosity (fraction of the moon illuminated combined with cloud coverage), and winter is time of winter (number of days since December 1st). Wind, snow and luminosity are scaled by mean-centering and then being divided by their standard deviations. | | | | |
| --- | --- | --- | --- | --- |
| **Fixed effect** | **Estimate** | **SE** | **t value** | **p-value** |
| Intercept | -1.18 | 0.57 | -2.06 | 0.04 |
| Wind (scaled) | -0.03 | 0.07 | -0.48 | 0.63 |
| Snow (scaled) | -0.02 | 0.08 | -0.23 | 0.82 |
| Luminosity (scaled) | -0.03 | 0.08 | -0.43 | 0.67 |
| Winter | 0.02 | 0.01 | 4.13 | <0.001 |
| Sex: male | 2.60 | 1.07 | 2.42 | 0.02 |
| Winter*Sex: M | -0.02 | 0.01 | -1.54 | 0.14 |
| **Random effect** | **Variance** | **SD** |  |  |
| Porcupine ID | 0.7186 | 0.8477 |  |  |
| Residual | 0.9493 | 0.9743 |  |  |
| **Conditional R^2^** | 0.54 |  |  |  |
| **Marginal R^2^** | 0.19 |  |  |  |

| Table S9. Comparison of linear mixed effects models with and without body mass of the individual (kg) upon capture, and parameter estimates from the model with body mass as an additive effect, explaining the deviations of observed activity time of North American porcupines *Erethizon dorsatum* from the prediction by the energetics-based model for T*a* > T*LC*. Wind is the mean daily wind speed, snow is the daily snow depth, luminosity is lunar luminosity (fraction of the moon illuminated combined with cloud coverage), and winter is time of winter (number of days since December 1st). Wind, snow and luminosity are scaled by mean-centering and then being divided by their standard deviations. *df* is degree of freedom, and AICc is Akaike information criterion for small sample size. | | | | |
| --- | --- | --- | --- | --- |
| **Model** | ***df*** | **∆AICc** | **AICc weight** | |
| wind + snow + luminosity + winter*sex + (1\|ID) | 9 | 0 | 0.477 | |
| wind + snow + luminosity + body mass + winter*sex + (1\|ID) | 10 | 0.44 | 0.383 | |
| wind + snow + luminosity + winter*body mass + winter*sex + (1\|ID) | 11 | 2.44 | 0.141 | |
| **Fixed effect** | **Estimate** | **SE** | **t value** | **p-value** |
| Intercept | -1.11 | 1.19 | -0.93 | 0.36 |
| Wind (scaled) | -0.03 | 0.07 | -0.50 | 0.62 |
| Snow (scaled) | -0.03 | 0.08 | -0.33 | 0.74 |
| Luminosity (scaled) | -0.03 | 0.08 | -0.41 | 0.68 |
| Body mass | -0.20 | 0.17 | -1.16 | 0.26 |
| Winter | 0.02 | 0.01 | 3.77 | <0.001 |
| Sex: male | 2.21 | 1.14 | 1.94 | 0.06 |
| Winter*Sex: M | -0.01 | 0.01 | -1.05 | 0.30 |
| **Random effect** | **Variance** | **SD** |  |  |
| Porcupine ID | 0.7003 | 0.8368 |  |  |
| Residual | 0.9516 | 0.9755 |  |  |
| **Conditional R^2^** | 0.56 |  |  |  |
| **Marginal R^2^** | 0.23 |  |  |  |


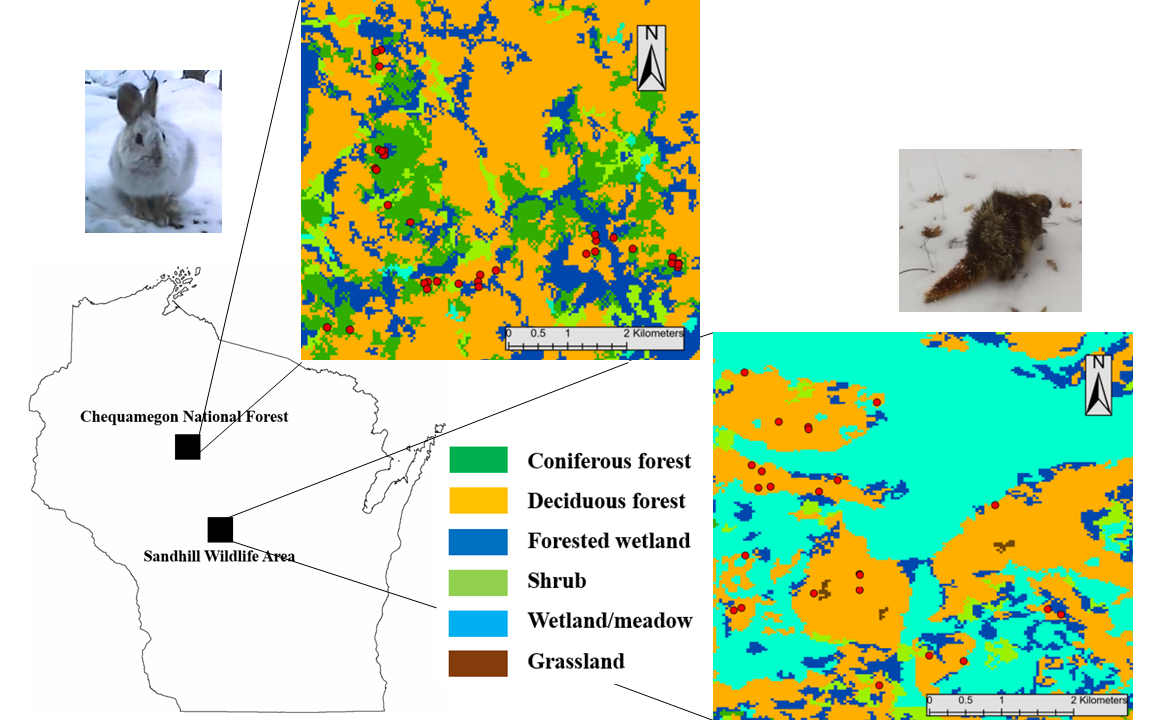


Figure S1. Map of the monitoring areas for snowshoe hares *Lepus americanus* in Chequamegon National Forest, Wisconsin, and for North American porcupines *Erethizon dorsatum* in Sandhill Wildlife Area, Wisconsin, USA, overlaid with cover types from Wiscland 2 (Wisconsin Department of Natural Resources 2016). Red circles represent the capture locations of the hares and porcupines deployed with accelerometers in winters 2022-2023.


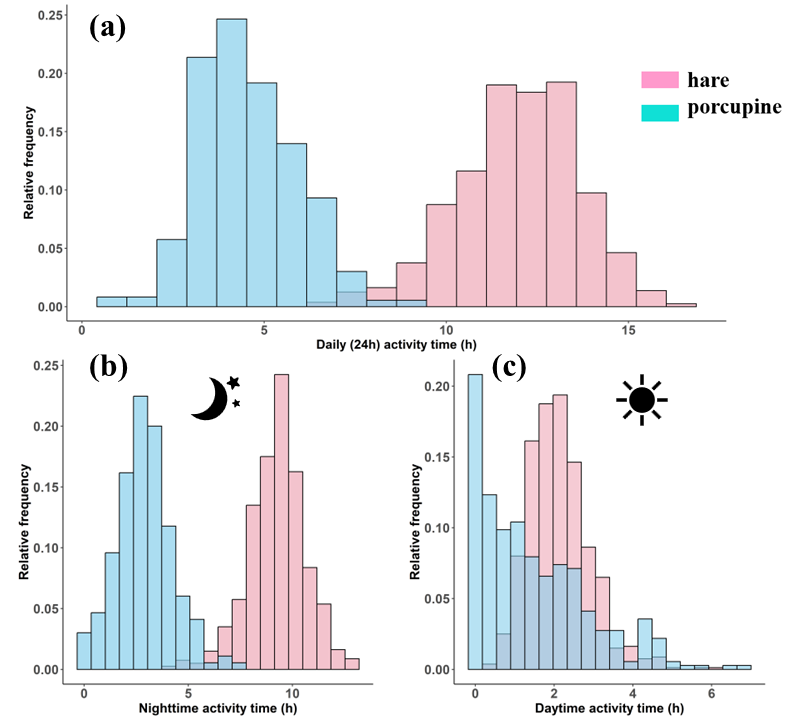


Figure S2. Distribution of daily (over 24 h) (a), nighttime (b) and daytime (c) activity time of snowshoe hares *Lepus americanus* and North American porcupines *Erethizon dorsatum* in central Wisconsin, USA in winter 2022-2023.


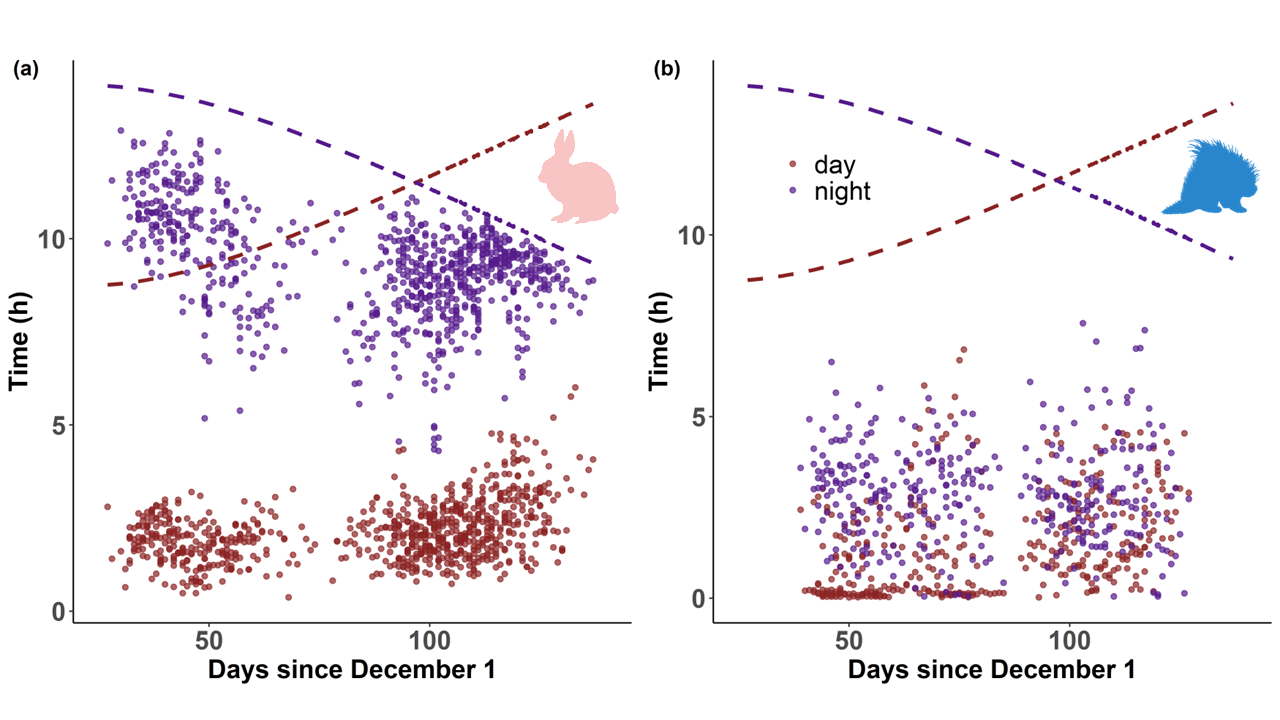


Figure S3. Observed daytime and nighttime activity time of (a) snowshoe hares *Lepus americanus* and (b) North American porcupines *Erethizon dorsatum* in central Wisconsin, USA over the winter shown with the changes in day length (brown dotted line) and in night length (purple dotted line).


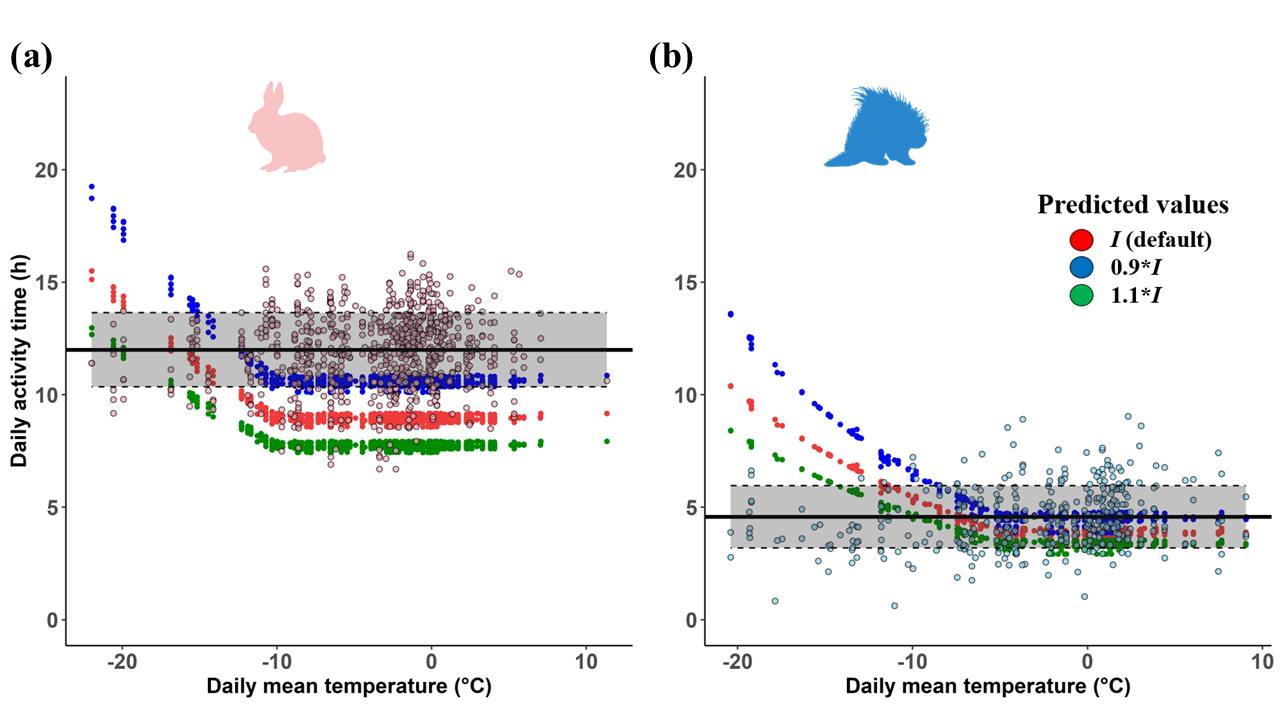


Figure S4. Comparison of predicted and observed daily activity time for hares *Lepus americanus* (a) and porcupines *Erethizon dorsatum* (b) in central Wisconsin, USA. In each graph, the daily activity time in winter predicted by the energetics-based activity model is shown for three levels of energy intake (red points: predicted values using the default energy intake rate *I* shown in Table S2, blue points: predicted values when *I* is reduced by 10 %, green points: predicted values when *I* is increased by 10 %). The observed daily activity time is presented with unique points for each species (black circles filled with flesh-tone for snowshoe hares (a) and black circles filled with light blue for porcupines (b)). Solid horizontal lines represent the means of observed daily activity time and dotted horizontal lines represent the mean ± standard deviation of the observed activity time, for each species.


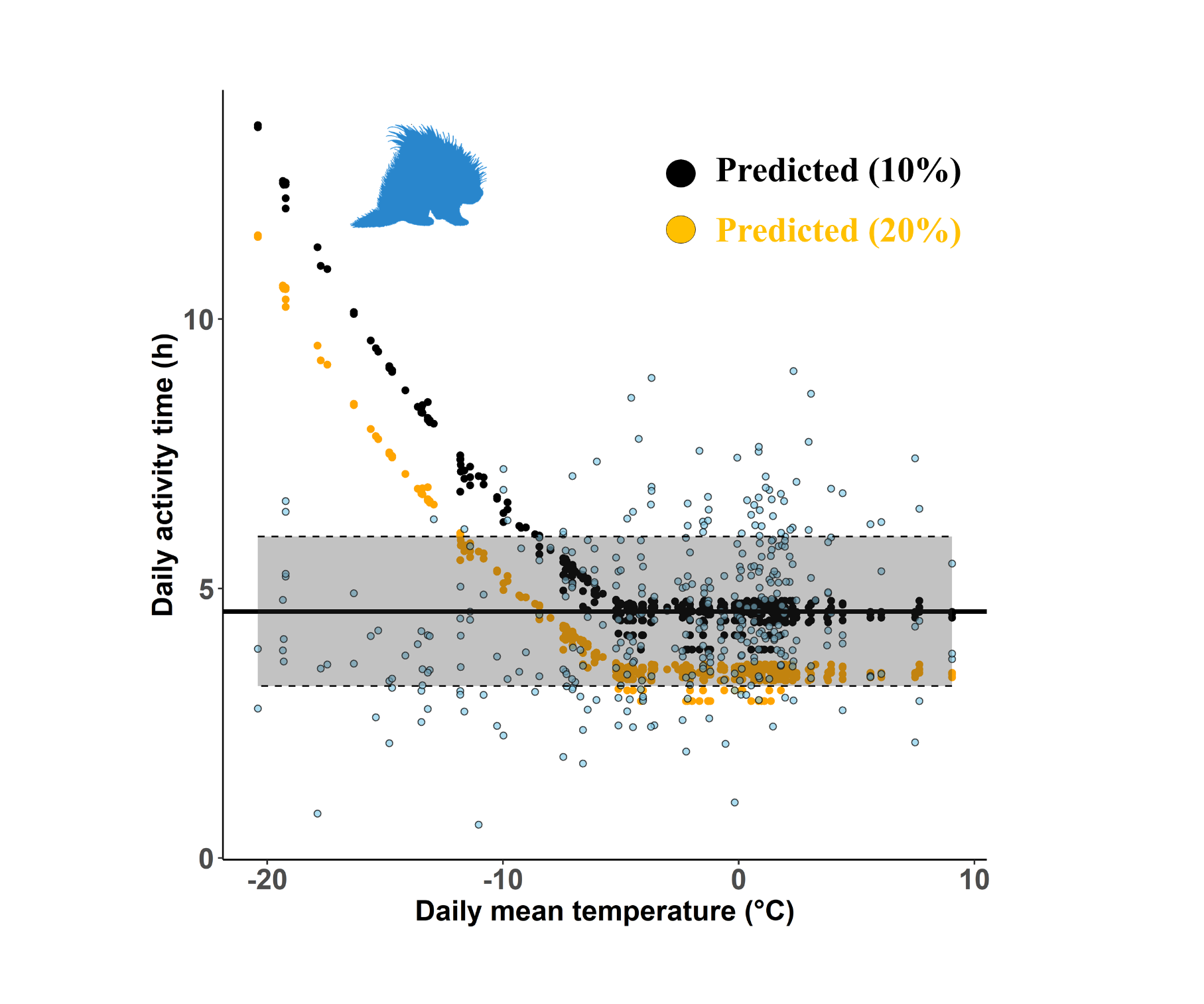


Figure S5. Daily activity time in winter predicted for North American porcupines *Erethizon dorsatum* in central Wisconsin, USA, by the energetics-based activity model, presented with observed activity time. Predictions were made assuming that either 10 % (black points) or 20 % (orange points) of their daily energy requirement would be drawn from fat catabolism. Smaller points (faded blue) are the observed values, solid horizontal lines represent the means of observed activity time, and dotted horizontal lines represent the mean ± standard deviation of the observed activity time.


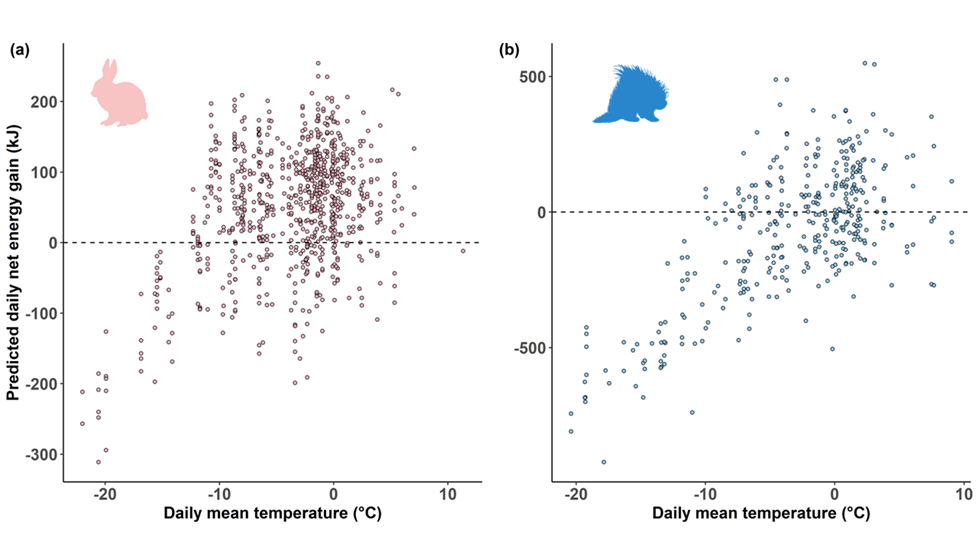


Figure S6. Daily net energy gain (kJ) predicted by the energetics-based model based on observed daily activity time and daily temperature for (a) snowshoe hares *Lepus americanus* and (b) in North American porcupines *Erethizon dorsatum* in central Wisconsin, USA.

**References for Supplementary Materials**

Clauss, M., Schwarm, A., Ortmann, S., Streich, W. J., & Hummel, J. (2007). A case of non-scaling in mammalian physiology? Body size, digestive capacity, food intake, and ingesta passage in mammalian herbivores. *Comparative Biochemistry and Physiology Part A: Molecular & Integrative Physiology*, *148*(2), 249-265.

Coltrane, J. A., & Barboza, P. S. (2010). Winter as a nutritional bottleneck for North American porcupines (Erethizon dorsatum). *Journal of Comparative Physiology B*, *180*, 905-918.

Coltrane, J. A., Farley, S., Barboza, P. S., Kohl, F., Sinnott, R., & Barnes, B. M. (2011). Seasonal body composition, water turnover, and field metabolic rates in porcupines (Erethizon dorsatum) in Alaska. *Journal of Mammalogy*, *92*(3), 601-610.

Coltrane, J. A., & Sinnott, R. (2013). Winter home range and habitat use by porcupines in Alaska. *The Journal of wildlife management*, *77*(3), 505-513.

DeMatteo, K. E., & Harlow, H. J. (1997). Thermoregulatory responses of the North American porcupine (Erethizon dorsatum bruneri) to decreasing ambient temperature and increasing wind speed. *Comparative Biochemistry and Physiology Part B: Biochemistry and Molecular Biology*, *116*(3), 339-346.

Ellsworth, E., Boudreau, M. R., Nagy, K., Rachlow, J. L., & Murray, D. L. (2016). Differential sex-related winter energetics in free-ranging snowshoe hares (Lepus americanus). *Canadian Journal of Zoology*, *94*(2), 115-121.

Fournier, F., & Thomas, D. W. (1999). Thermoregulation and repeatability of oxygen-consumption measurements in winter-acclimatized North American porcupines (Erethizon dorsatum). *Canadian Journal of Zoology*, *77*(2), 194-202.

Fristoe, T. S., Burger, J. R., Balk, M. A., Khaliq, I., Hof, C., & Brown, J. H. (2015). Metabolic heat production and thermal conductance are mass-independent adaptations to thermal environment in birds and mammals. *Proceedings of the National Academy of Sciences*, *112*(52), 15934-15939.

Humphries, M. M., & Umbanhowar, J. (2007). Filtering environmental variability: activity optimization, thermal refuges, and the energetic responses of endotherms to temperature. In *The Impact of Environmental Variability on Ecological Systems* (pp. 61-87). Dordrecht: Springer Netherlands.

Karasov, W. H. (1992). Daily energy expenditure and the cost of activity in mammals. *American Zoologist*, *32*(2), 238-248.

Mabille, G., Berteaux, D., Thomas, D. W., & Fortin, D. (2011). Behavioural responses of wintering porcupines to their heterogeneous thermal environment. *Ecoscience*, *18*(4), 341-353.

Martin, M. E., Moriarty, K. M., & Pauli, J. N. (2020). Forest structure and snow depth alter the movement patterns and subsequent expenditures of a forest carnivore, the Pacific marten. *Oikos*, *129*(3), 356-366.

Montevecchi, W. A., Birt-Friesen, V. L., & Cairns, D. K. (1992). Reproductive energetics and prey harvest of Leach's storm‐petrels in the northwest Atlantic. *Ecology*, *73*(3), 823-832.

Resheff, Y. S., Rotics, S., Harel, R., Spiegel, O., & Nathan, R. (2014). AcceleRater: a web application for supervised learning of behavioral modes from acceleration measurements. *Movement ecology*, *2*, 1-7.

Riek, A., & Geiser, F. (2013). Allometry of thermal variables in mammals: consequences of body size and phylogeny. *Biological reviews*, *88*(3), 564-572.

Roze, U. (2009). *The North American porcupine*. Cornell University Press.

Savage, V. M., Gillooly, J. F., Woodruff, W. H., West, G. B., Allen, A. P., Enquist, B. J., & Brown, J. H. (2004). The predominance of quarter‐power scaling in biology. *Functional Ecology*, *18*(2), 257-282.

Sheriff, M. J., Kuchel, L., Boutin, S., & Humphries, M. M. (2009). Seasonal metabolic acclimatization in a northern population of free-ranging snowshoe hares, Lepus americanus. *Journal of Mammalogy*, *90*(3), 761-767.

Studd, E. K., Boudreau, M. R., Majchrzak, Y. N., Menzies, A. K., Peers, M. J., Seguin, J. L., ... & Humphries, M. M. (2019). Use of acceleration and acoustics to classify behavior, generate time budgets, and evaluate responses to moonlight in free-ranging snowshoe hares. *Frontiers in Ecology and Evolution*, *7*, 154.

Studd, E. K., Menzies, A. K., Siracusa, E. R., Dantzer, B., Lane, J. E., McAdam, A. G., ... & Humphries, M. M. (2020). Optimisation of energetic and reproductive gains explains behavioural responses to environmental variation across seasons and years. *Ecology Letters*, *23*(5), 841-850.

Studd, E. K., Peers, M. J. L., Menzies, A. K., Derbyshire, R., Majchrzak, Y. N., Seguin, J. L., ... & Boutin, S. (2022). Behavioural adjustments of predators and prey to wind speed in the boreal forest. *Oecologia*, *200*(3), 349-358.

Sullender, B. K., Cunningham, C. X., Lundquist, J. D., & Prugh, L. R. (2023). Defining the danger zone: critical snow properties for predator–prey interactions. *Oikos*, *2023*(10), e09925.

Thieurmel B, Elmarhraoui A (2022). _suncalc: Compute Sun Position, Sunlight Phases, Moon Position and Lunar Phase_. R package version 0.5.1, <https://CRAN.R-project.org/package=suncalc>.

Wisconsin Department of Natural Resources. 2016. Wiscland 2 land cover user guide. <https://dnr.wisconsin.gov/maps/WISCLAND>.
